# Putting psychological interventions first in primary health care

**DOI:** 10.1002/wps.21114

**Published:** 2023-09-15

**Authors:** Mark van Ommeren, Sian Lewis, Edith van't Hof, Kenneth Carswell

**Affiliations:** ^1^ Department of Mental Health and Substance Use World Health Organization Geneva Switzerland

Task‐sharing – in which specialists train, supervise and support non‐specialist health care providers – is proven to be acceptable, feasible and effective in scaling up mental health care for depressive and anxiety disorders^1^. In this perspective, we focus on reasons for and barriers to task‐sharing of psychological interventions in primary health care. We also cover what the World Health Organization (WHO) does to address these barriers.

Task‐sharing in primary health care is vital to increase treatment coverage for people in need, but it rarely includes providing evidence‐based psychological interventions. Yet research shows that cognitive‐behavioral therapy (CBT), on its own or combined with antidepressants, is the first‐line treatment for adult depressive disorders^2^. CBT is also first‐line treatment for other conditions, including anxiety disorders. Several other psychological therapies – such as interpersonal, problem solving and behavioral activation therapies – are likely equally effective^3^.

Many evidence‐based psychological interventions are well suited to task‐sharing. They can be designed to be safely delivered by supervised non‐specialists. They can be adjusted to be briefer and less resource‐intensive than conventional psychotherapy, without being less effective^1^. And they can be adapted for remote or group delivery or provided through guided or unguided self‐help manuals, websites and applications. WHO's Problem Management Plus, for example, comprises just five weekly sessions, can be delivered to individuals or groups, and is suitable for many contexts, types of adversity and types of helpers^4^.

Despite their potential, psychological interventions are rarely provided at scale^5^. Yet scale up is possible. The National Mental Health Programme in Lebanon is showing that implementing a nationwide self‐help intervention for depression is feasible, even amid multiple crises^1^, ^6^.

There are many barriers to including psychological interventions in task‐sharing:

- *Lack of political support*. Despite the evidence, decision‐makers in many countries remain unaware of the effectiveness of psychological interventions and so exclude them from universal health coverage packages of essential services and financial protection schemes.
- *Resistance to change*. Still some psychologists today – including some national psychological associations – are against sharing responsibility for delivering psychological treatments with non‐specialists. The reality though is that no society, however rich, will ever have enough specialists to offer more than a fraction of the volume of care required to help the large numbers of people who need mental health interventions.
- *Little commercial incentive*. Despite their cost‐effectiveness, there is little commercial incentive to make psychological interventions widely available. By comparison, pharmacological interventions are heavily promoted by pharmaceutical companies, which may influence decision‐makers and medical staff to focus on drug treatments^6^.
- *Lack of human resources*. Task‐sharing for psychological interventions in primary health care typically means recruiting and retaining additional (non‐specialist, community‐based) staff to deliver those interventions. This is needed since medical staff in primary health care typically have heavy workloads and, while they can refer people for psychological interventions, they rarely have time to deliver lengthy therapeutic sessions themselves.
- *Lack of financial resources*. Funding a national workforce of providers, trainers and supervisors demands larger mental health budgets than are currently available. This means that more funds must be allocated within health budgets or, importantly, from the state treasury.
- *Lack of access to relevant tools*. Too few proven psychological intervention manuals for non‐specialists are freely available (open access)^7^.
- *Lack of operational guidance*. Apart from the Design, Implementation, Monitoring and Evaluation (DIME) manuals^8^, there is little international guidance on how to integrate psychological interventions in primary health care. Even if service planners want to add those interventions to their services, they may not know what steps, service models and resources they need.

Building on the work of many others, the WHO is addressing a range of these barriers. We recommend psychological interventions and promote task‐sharing through our *Comprehensive Mental Health Action Plan 2013‐2030*, our mhGAP programme, our Universal Health Coverage (UHC) compendium and our *World Mental Health Report* ^1^. We develop, test and publish open access diverse psychological interventions that are scalable and suit different delivery models. And we support training and supervision tools to help assure a competent non‐specialist workforce through our Ensuring Quality in Psychological Support (EQUIP) initiative^9^.

We are also finalizing a new, operational guide – a *Psychological Interventions Implementation Manual* – to help service planners and programmers add psychological interventions to their services. Written for managers and others responsible for planning and implementing services, this manual provides practical guidance on how to plan, prepare and provide psychological interventions within existing services, such as health, social or education services.

This new WHO manual advises service planners on how to: a) choose and adapt psychological interventions to be relevant for their specific settings; b) decide a setting and system for delivery, including linking to associated services; c) develop a competent workforce by selecting, training, assessing and supervising providers; d) identify potential service users, assess their support needs and ensure people get the care they need; and e) use monitoring and evaluation to evaluate and improve the service provided.

The manual marks the latest addition to our toolbox for psychological interventions. After publication, it will be field‐tested and refined.

Service planners can now freely access all the resources they need to implement psychological interventions: intervention manuals, tools to support competence, and operational guidance for implementation. The next big step is to get these resources into use. Ultimately, this work is intended to help improve the quality and local availability of evidence‐based mental health care, so that millions more people with depression and anxiety will be effectively helped.
